# Effect of birthplace on cardiometabolic risk among blacks in the Metabolic Syndrome Outcome Study (MetSO)

**DOI:** 10.1186/s13098-016-0130-z

**Published:** 2016-02-24

**Authors:** Joseph Ravenell, Azizi Seixas, Diana Margot Rosenthal, Olajide Williams, Chinwe Ogedegbe, Mary Ann Sevick, Valerie Newsome, Girardin Jean-Louis

**Affiliations:** Department of Population Health, Center for Healthful Behavior Change (CHBC), New York University Medical Center, 227 East 30th Street (between 2nd and 3rd Ave), Floor # 6-629D, New York, NY 10016 USA; Department of Neurology, Columbia University Medical Center, New York, NY USA; Department of Emergency Medicine, Hackensack UMC, Hackensack, NJ USA

**Keywords:** Place of birth, Cardiometabolic profile, Metabolic syndrome

## Abstract

**Background:**

Metabolic syndrome poses an increased global burden of disease and causes immense financial burden, warranting heightened public health attention. The present study assessed the prevalence and severity of cardiometabolic risk among foreign-born versus US-born blacks, while exploring potential gender-based effects.

**Methods:**

A total of 1035 patients from the Metabolic Syndrome Outcome Study (Trial registration: NCT01946659) provided sociodemographic, medical history, and clinical data. General Linear Model (GLM) was used to assess the effects of birthplace and gender on cardiometabolic parameters, adjusting for age differences in the sample.

**Results:**

Of the sample, 61.6 % were foreign-born blacks (FBB) and 38.4 % were US-born blacks (USB). FBB had significantly lower BMI compared with USB (32.76 ± 0.35 vs. 35.41 ± 0.44, F = 22.57), but had significantly higher systolic blood pressure (136.70 ± 0.77 vs. 132.83 ± 0.98; F = 9.60) and fasting glucose levels than did USB (146.46 ± 3.37 vs. 135.02 ± 4.27; F = 4.40). Men had higher diastolic BP (76.67 ± 0.65 vs. 75.05 ± 0.45; F = 4.20), glucose (146.53 ± 4.48 vs. 134.95 ± 3.07; F = 4.55) and triglyceride levels (148.10 ± 4.51 vs. 130.60 ± 3.09; F = 10.25) compared with women, but women had higher LDL-cholesterol (109.24 ± 1.49 vs. 98.49 ± 2.18; F = 16.60) and HDL-cholesterol levels (50.71 ± 0.66 vs. 42.77 ± 0.97; F = 46.01) than did men.

**Conclusions:**

Results showed that birthplace has a significant influence on cardiometabolic profiles of blacks with metabolic syndrome. Patients’ gender also had an independent influence on cardiometabolic profile.

## Background

Metabolic syndrome (MetS), a constellation of medical conditions that predispose individuals to increased risk of cardiovascular disease (CVD), Type 2 diabetes mellitus (T2DM) and all-cause mortality [1], increases global burden of disease and causes immense financial burden, warranting heightened public health attention [2–7]. MetS components include hyperglycemia (fasting glucose ≥100 mg/dL), central or abdominal obesity (measured by waist circumference ≥40 inches for men and ≥35 inches for women), elevated blood pressure (systolic/diastolic blood pressure ≥130/85 mmHG), and atherogenic dyslipidemia (triglyceride levels ≥150 mg/dL of blood and HDL cholesterol <40 mg/dL in men and <50 mg/dL in women). MetS risk is greater among older adults and seems more prevalent among blacks relative to whites, although this may be gender dependent [8].

Among blacks, sociodemographic and environmental factors appear to be associated with the presence of MetS or its components. Using the Jackson Heart Study, researchers found that metabolic syndrome was positively correlated with neighborhood socioeconomic disadvantage, and lack of perceived safety was associated with elevated glucose and waist circumference [9]. An intriguing hypothesis has emerged suggesting that place of birth or geographic residence could influence prevalence and severity of metabolic syndrome as well as its associated costs [10]. Evidence suggests an increase in metabolic syndrome related hospitalizations may be attributed in part to places of residence replete with persistent environmental and organic pollutants that induce insulin resistance. Furthermore, since a disproportionate number of blacks are born and/or reside in zip codes characterized by persistent environmental and organic pollutants relative to whites, it is likely that place of birth and residence contribute to poor cardiometabolic health [10].

We previously examined cardiometabolic risk profiles of US-born blacks (USB) versus foreign-born blacks (FBB), finding that higher rates of dyslipidemia and diabetes exist among USB relative to FBB [11]. However, detailed analyses of potential effects of place of birth on metabolic syndrome and its components could not be performed because of limited sample size, lack of clinical metabolic syndrome data, and restricted age range in that study. The current study builds on our previous work, examining effects of place of birth on cardiometabolic risks among blacks in the Metabolic Syndrome Outcome Study (MetSO). Specifically, we assessed the prevalence and severity of cardiometabolic risk factors in foreign-born versus US-born blacks, while exploring potential gender-based effects.

## Methods

### Patient population

Data (n = 1035) for the present investigation emanated from the MetSO study, which investigated sociodemographic, behavioral, psychosocial factors as well as health risks associated with metabolic syndrome among blacks in the primary-care setting. Data were obtained from four primary-care clinics in Brooklyn, NY affiliated with SUNY Downstate Medical Center.

### Procedures

Study staff approached patients in primary-care clinics to solicit their participation. Those agreeing to participate provided informed consent and filled out a battery of tests including a questionnaire on sociodemographic profile, medical history, and use of medications. Clinical data including body mass index (BMI), blood pressure (BP), high-density lipoprotein cholesterol (HDL), low-density lipoprotein cholesterol (LDL), and fasting plasma glucose (FPG) or hemoglobin (HbA1c) for those who had a diagnosis of diabetes were obtained from an electronic medical record at SUNY Downstate Medical Center, where all participants were initially registered [12]. The Institutional Review Boards (IRB) at New York University Langone Medical Center and SUNY Downstate Medical Center approved this study.

### Statistical analysis

Frequency and measures of central tendency were used to describe the sample. In preliminary analyses, Pearson and Spearman correlations were used to explore relationships between variables of interest. General Linear Model (GLM) was used to assess the effects of birthplace and gender on cardiometabolic parameters, adjusting for age differences in the sample. In preliminary analyses, income, education, and health risks did not have significant co-varying effects on the dependent measures. Thus, only age effects were adjusted in the final model. The model also ascertained whether birthplace and gender had a significant interaction with cardiometabolic factors. All analyses were performed using SPSS (version 19.0; SPSS Inc. Chicago) statistical software.

## Results

Of the 1035 patients, 986 reported their birthplace: 607 (61.6 %) were FBB and 379 (38.4 %) were USB. Sociodemographic characteristics are reported in Table 1. Compared to USB, FBB patients were older and more likely to be married. FBB were less likely to have a high school diploma and more likely to report an annual household income less than $10,000 compared to USB. Smoking rates were strikingly dissimilar, with USB reporting a smoking rate 2.5 times higher than FBB.

**Table 1:** Sociodemographic and health risks among blacks in the MetSO cohort (foreign-born blacks and US-born blacks)

Variables	FBB	USB	Total sample
Age (years)	63.04	60.94	62.24
Sex (female) %	69.1	68.1	68.7
Marital status %			
Married	37.0	23.6	31.8
Widowed	15.1	20.0	17.0
Divorced	17.4	12.6	15.5
Separated	10.6	9.0	10.0
Never married	19.4	34.2	25.2
Education (< HS) %	38.7	32.1	36.2
Family income (<10K)	45.5	40.3	43.5
Smoking history (yes) %	19.0	47.1	29.2
Drinking (yes) %	20.4	22.2	21.1

Smoking history, “Have you ever had more than 100 cigarettes ever”

Drinking, “In the past 30 days have you had at least one drink?”

Prevalence of physician diagnosed-conditions in the study sample is reported in Table 2. Descriptive analysis showed that FBB and USB had similar rates of hypertension, dyslipidemia and stroke. FBB had significantly lower rates of obesity (66.4 vs.74.9 %) and self-reported diagnosis of heart disease (26.5 vs. 37.5 %) compared to USB. However, FBB had significantly higher rates of diabetes (63.1 vs. 55.7 %) than USB.

**Table 2:** Differences in physician-diagnosed medical conditions among blacks in the MetSO cohort based on birthplace (foreign-born blacks and US-born blacks)

Variable	FBB	USB	X^2^	*p*
Hypertension %	91.9	92.6	0.182	0.670
Dyslipidemia %	75.6	71.7	1.757	0.185
Diabetes %	63.1	55.7	5.172	0.023*
Obesity %	66.4	74.9	9.802	0.007**
Heart disease %	26.5	37.5	12.885	<0.001***
Stroke %	8.3	10.1	0.859	0.354

Hypertension, BP >140 mmHg; dyslipidemia, total cholesterol >200 mg/dL ad triglycerides >150 mg.dL; diabetes, type 2 diabetes; obesity, BMI >30 kg/m^2^; heart disease, ever had a physician diagnosis or history of heart disease; stroke, ever had a physician diagnosis or history of stroke

* p < 0.05; ** *p* < 0.01; *** *p* < 0.001

Differences in individual cardiometabolic risk factors and components of metabolic syndrome are reported by birthplace (Table 3) and gender (Table 4). While there were no birthplace-based differences in lipid profile, HbA1c, and diastolic blood pressure, there were significant between-group differences in BMI, systolic blood pressure, and fasting glucose levels. FBB had significantly lower mean BMI compared to USB (32.76 vs. 35.41 kg/m^2^), but had significantly higher systolic blood pressure (136.70 vs. 132.83 mmHg) and fasting glucose (146.46 vs. 135.02 mg/dL) levels than USB. Gender effect analyses showed that there were no significant differences between men and women regarding BMI, systolic BP, or HbA1c. However, there were significant gender effects on diastolic BP, fasting glucose, and components of the lipid profile (see Table 4). Men had higher diastolic BP, glucose, and triglyceride levels compared to women, but women had higher LDL-cholesterol and HDL-cholesterol levels (see Table 4).

**Table 3:** Differences in cardiometabolic risk profile based on birthplace among blacks with metabolic syndrome

Variable	FBB (Mean ± SE)	USB (Mean ± SE)	F	*p*
BMI	32.76 ± 0.35	35.41 ± 0.44	22.57	<0.001***
Systolic BP	136.70 ± 0.77	132.83 ± 0.98	9.60	0.002**
Diastolic BP	76.54 ± 0.49	75.19 ± 0.62	2.91	0.088
Glucose	146.46 ± 3.37	135.02 ± 4.27	4.40	0.036*
LDL	104.91 ± 1.64	102.81 ± 2.08	0.62	0.430
HDL	47.00 ± 0.73	46.48 ± 0.92	0.19	0.661
Triglycerides	137.33 ± 3.40	141.36 ± 4.30	0.54	0.464
Hba1c	8.95 ± 0.48	8.15 ± 0.60	1.08	0.299

*BMI* body mass index, *systolic BP* systolic blood pressure, *diastolic BP* diastolic blood pressure, *LDL* low-density lipoprotein, *HDL* high-density lipoprotein, *Hba1c* glycated hemoglobin

* p < 0.05; ** *p* < 0.01; *** p < 0.001

**Table 4:** Gender-based differences in cardiometabolic risk profile among blacks with metabolic syndrome, adjusting for age differences

Variable	Men (mean ± SE)	Women (mean ± SE)	F	*p*
BMI	33.93 ± 0.46	34.24 ± 0.32	0.31	0.579
Systolic BP	135.22 ± 1.02	134.31 ± 0.70	0.53	0.466
Diastolic BP	76.67 ± 0.65	75.05 ± 0.45	4.20	0.041*
Glucose	146.53 ± 4.48	134.95 ± 3.07	4.55	0.033*
LDL	98.49 ± 2.18	109.24 ± 1.49	16.60	<0.001***
HDL	42.77 ± 0.97	50.71 ± 0.66	46.01	<0.001***
Triglycerides	148.10 ± 4.51	130.60 ± 3.09	10.25	0.001**
Hba1c	8.31 ± 0.63	8.78 ± 0.43	0.38	0.539

*BMI* body mass index, *systolic BP* systolic blood pressure, *diastolic BP* diastolic blood pressure, *LDL* low-density lipoprotein, *HDL* high-density lipoprotein, *HbA1c* glycated hemoglobin

* *p* < 0.05; ** *p* < 0.01; *** p < 0.001

## Discussion

In this cross-sectional analysis of 1035 FBB and USB with metabolic syndrome in New York city, we found that rates of hypertension, dyslipidemia, and stroke did not differ by birthplace, which is consistent with previous studies. Likewise, we found that FBB had lower rates of obesity, heart disease, and smoking behavior relative to USB. However, critical cardiovascular risk factors, such as mean systolic blood pressure and rates of diabetes, were significantly higher among FBB compared to USB. This is a novel and unexpected finding in light of previous studies, including our own, that have demonstrated similar or lower cardiovascular risk among FBB compared to USB.

The finding that FBB had higher rates of diabetes is remarkable given that FBB, in this study, were less likely to be obese, and had significantly lower mean BMI compared to USB. Our finding that FBB had higher rates of diabetes and were less likely to be obese compared to USB is incongruent with well-established evidence that higher BMI, obesity, and diabetes risk are positively associated. One possible explanation is that FBB may differ in visceral adipose tissue (VAT) compared to USB, despite having lower BMI [13]. Visceral adipose tissue is thought to be an important determinant of metabolic risk, and may be more predictive of metabolic risk than BMI. Previous studies have found that VAT and BMI do not necessarily correlate, particularly among blacks [13]. Thus, it is conceivable that FBB may have a more deleterious pattern of fat distribution despite a lower BMI, which puts them at higher risk of diabetes compared to USB. Thus, one area of future research may be to examine differences in VAT between FBB and USB, and the underlying biologic and psychosocial mechanisms for these potential differences.

Another point that warrants discussion is the higher systolic BP among FBB compared to USB in our study. This finding is slightly contrary to the east to west blood pressure gradient hypothesis described by Richard Cooper and colleagues (1997). This hypothesis states that blacks in eastern countries have lower hypertension rates compared to blacks in western countries, suggesting that industrialization, which is more common in western countries, might be responsible for variability in hypertension rates [14]. One important consideration in interpreting the finding of a higher mean SBP among FBB is acculturation and duration of stay in the United States. It has been previously demonstrated that while FBB have better health status than USB for several indicators (e.g. self-rated health, health related quality of life, hypertension, diabetes and obesity) [15, 16], duration of stay in the US greater than 4 years among FBB was associated with worse health outcomes, comparable to the health status of USB [17]. Borrell et al. found that the prevalence of self-reported hypertension in FBB actually exceeded the prevalence of self-reported hypertension in USB, but only among FBB who had lived in the US for 10 years or more [16]. These findings, reinforced by the results of the current study, suggest that there may be factors for FBB that worsen health after spending time in the U.S. Such a possibility calls for further population-based investigation to understand the widening disparity in cardiovascular outcomes among FBB residing in the US as well as development of population-specific interventions We also found a significant gender effect on cardiometabolic risk in our diverse sample of black participants after controlling for age. Specifically, there were gender-based differences in fasting glucose levels and lipid profile. Our finding of higher glucose levels among men is consistent with other large–scale studies such as NHANES and MESA [18], which found similar gender-based differences in glucose levels. This gender-based trend is also consistent with findings from the Jackson Heart Study, a longitudinal cohort study of US blacks, which consistently showed lower fasting glucose levels among women, regardless of glucose classification (normal, impaired fasting glucose, and diabetes) [19]. With regard to lipids, women had higher LDL and HDL levels, but lower triglyceride levels. Other studies have found, including Multi-Ethnic Study of Atherosclerosis (MESA) [20] and the Jackson Heart Study [21], that black women are less likely to have a dyslipidemic profile (low HDL and high triglycerides) than other groups. In fact, it has been proposed that this tendency to have a more favorable lipid profile may undermine cardiovascular risk assessment in black women, particularly among women with metabolic syndrome [22, 23]. A recent study by McIntosh et al. found that among a sample of 254 women of which 116 were black, only less than 1 % met criteria for dyslipidemia [24]. Yet, black women have high rates of obesity, hypertension and Type 2 diabetes, thus increasing their cardiovascular risk, despite having a more favorable lipid profile. These factors should be taken into account in cardiovascular risk assessment and stratification for black women, particularly among those with metabolic syndrome.

Despite the strengths of having a robust number of foreign-born and US-born blacks in our sample, our study has some limitations. First, we did not collect information on dietary practices, such as eating habits and food intake, or family history of hypertension and diabetes—all critical cardiovascular risk factors. Family history may provide insight on the noted differences in blood pressure and diabetes between FBB and USB. Second, information on country of birth, duration of stay and acculturation were not available for examination in this study. These and other psychosocial factors that may be unique to FBB may elucidate potential mechanisms for the higher blood pressure and diabetes risk noted in this population.

## Conclusion

In conclusion, we found that FBB have lower levels of obesity, similar rates of hypertension, dyslipidemia, stroke history, but higher rates of diabetes, history of heart disease, and higher systolic blood pressure compared to USB. These novel findings require further investigation and may have implications for addressing heath disparities in cardiovascular disease risk and outcomes.

## References

[1] Mendy VL, Azevedo MJ, Sarpong DF, Rosas SE, Ekundayo OT (2014). The association between individual and combined components of metabolic syndrome and chronic kidney disease among African Americans: the Jackson Heart Study. PLoS ONE.

[2] Ford ES, Giles WH, Dietz WH (2002). Prevalence of the metabolic syndrome among US adults: findings from the third National Health and Nutrition Examination Survey. JAMA.

[3] Moreira GC, Cipullo JP, Ciorlia LA, Cesarino CB, Vilela-Martin JF (2014). Prevalence of metabolic syndrome: association with risk factors and cardiovascular complications in an urban population. PLoS ONE.

[4] Liu M, Wang J, Jiang B, Sun D, Wu L (2013). Increasing prevalence of metabolic syndrome in a chinese elderly population: 2001–2010. PLoS ONE.

[5] Peer N, Lombard C, Steyn K, Levitt N. High prevalence of metabolic syndrome in the Black population of Cape Town: the cardiovascular risk in Black South Africans (CRIBSA) study. Eur J Prev Cardiol. 2014; pii: 2047487314549744. 10.1177/2047487314549744 25208906

[6] Mangat C, Goel NK, Walia DK, Agarwal N, Sharma MK (2010). Metabolic syndrome: a challenging health issue in highly urbanized union territory of north India. Diabetol Metab Syndr.

[7] Salas R, Bibiloni Mdel M, Ramos E, Villarreal JZ, Pons A (2014). Metabolic syndrome prevalence among Northern Mexican Adult population. PLoS ONE.

[8] Razzouk L, Muntner P (2009). Ethnic, gender, and age-related differences in patients with the metabolic syndrome. Curr Hypertens Rep.

[9] Clark CR, Ommerborn MJ, Hickson DA, Grooms KN, Sims M (2013). Neighborhood disadvantage, neighborhood safety and cardiometabolic risk factors in African Americans: biosocial associations in the Jackson Heart Study. PLoS ONE.

[10] Sergeev AV, Carpenter DO (2011). Increase in metabolic syndrome-related hospitalizations in relation to environmental sources of persistent organic pollutants. Int J Environ Res Public Health.

[11] Bamimore A, Olafiranye O, Demede M, Zizi F, Brown C (2012). High prevalence of hypertension and other cardiometabolic risk factors in US- and Caribbean-born blacks with chest pain syndromes. Cardiorenal Med..

[12] Williams NJ, Jean-Louis G, Brown CD, McFarlane SI, Boutin-Foster C (2014). Telephone-delivered behavioral intervention among blacks with sleep apnea and metabolic syndrome: study protocol for a randomized controlled trial. Trials.

[13] Araneta MR, Barrett-Connor E (2005). Ethnic differences in visceral adipose tissue and type 2 diabetes: Filipino, African-American, and white women. Obes Res.

[14] Cooper R, Rotimi C, Ataman S, McGee D, Osotimehin B (1997). The prevalence of hypertension in seven populations of west African origin. Am J Public Health..

[15] Read JG, Emerson MO, Tarlov A (2005). Implications of black immigrant health for U.S. racial disparities in health. J Immigr Health.

[16] Borrell LN, Crawford ND, Barrington DS, Maglo KN (2008). Black/white disparity in self-reported hypertension: the role of nativity status. J Health Care Poor Underserved.

[17] Fang J, Wheaton AG, Keenan NL, Greenlund KJ, Perry GS (2012). Association of sleep duration and hypertension among US adults varies by age and sex. Am J Hypertension.

[18] Menke A, Rust KF, Savage PJ, Cowie CC (2014). Hemoglobin A1c, fasting plasma glucose, and 2-h plasma glucose distributions in U.S. population subgroups: NHANES 2005–2010. Ann Epidemiol.

[19] Fox ER, Sarpong DF, Cook JC, Samdarshi TE, Nagarajarao HS (2011). The Relation of diabetes, impaired fasting blood glucose and insulin resistance to left ventricular structure and function in African Americans: the Jackson Heart Study. Diabet Care.

[20] Goff DC, Bertoni AG, Kramer H, Bonds D, Blumenthal RS (2006). Dyslipidemia prevalence, treatment, and control in the Multi-Ethnic Study of Atherosclerosis (MESA): gender, ethnicity, and coronary artery calcium. Circulation.

[21] Harman JL, Griswold ME, Jeffries NO, Sumner AE, Sarpong DF (2011). Age is positively associated with high-density lipoprotein cholesterol among African Americans in cross-sectional analysis: the Jackson Heart Study. J Clin Lipidol.

[22] Gaillard T, Schuster D, Osei K (2009). Metabolic syndrome in black people of the African diaspora the paradox of current classification, definition and criteria. Ethn Dis.

[23] Gaillard T, Schuster D, Osei K (2010). Differential impact of serum glucose, triglycerides, and high-density lipoprotein cholesterol on cardiovascular risk factor burden in nondiabetic, obese African American women: implications for the prevalence of metabolic syndrome. Metabolism.

[24] McIntosh MS, Kumar V, Kalynych C, Lott M, Hsi A (2013). Racial differences in blood lipids lead to underestimation of cardiovascular risk in black women in a nested observational study. Global Adv Health Med.

